# Fighting Cardiac Thromboembolism during Transcatheter Procedures: An Update on the Use of Cerebral Protection Devices in Cath Labs and EP Labs

**DOI:** 10.3390/life13091819

**Published:** 2023-08-28

**Authors:** Alberto Preda, Claudio Montalto, Michele Galasso, Andrea Munafò, Ilaria Garofani, Matteo Baroni, Lorenzo Gigli, Sara Vargiu, Marisa Varrenti, Giulia Colombo, Marco Carbonaro, Domenico Giovanni Della Rocca, Jacopo Oreglia, Patrizio Mazzone, Fabrizio Guarracini

**Affiliations:** 1 Electrophysiology Unit, De Gasperis Cardio Center, Niguarda Hospital, 20162 Milan, Italy; 2 Interventional Cardiology Unit, De Gasperis Cardio Center, Niguarda Hospital, 20162 Milan, Italy; claudio.montalto@ospedaleniguarda.it (C.M.); andreamuna1993@gmail.com (A.M.);; 3 Heart Rhythm Management Centre, Postgraduate Program in Cardiac Electrophysiology and Pacing, Universitair Ziekenhuis Brussel, Vrije Universiteit Brussel, European Reference Networks Guard-Heart, 1090 Brussels, Belgium; 4 Texas Cardiac Arrhythmia Institute, St. David’s Medical Center, Austin, TX 78705, USA; 5 Department of Cardiology, Santa Chiara Hospital, 38122 Trento, Italy

**Keywords:** cerebral protection, cerebral protection devices, left atrial appendage closure, ventricular tachycardia ablation, transcatheter procedures, stroke

## Abstract

Intraprocedural stroke is a well-documented and feared potential risk of cardiovascular transcatheter procedures (TPs). Moreover, subclinical neurological events or covert central nervous system infarctions are concerns related to the development of dementia, future stroke, cognitive decline, and increased risk of mortality. Cerebral protection devices (CPDs) were developed to mitigate the risk of cardioembolic embolism during TPs. They are mechanical barriers designed to cover the ostium of the supra-aortic branches in the aortic arch, but newer devices are able to protect the descending aorta. CPDs have been mainly designed and tested to provide cerebral protection during transcatheter aortic valve replacement (TAVR), but their use in both Catheterization and Electrophysiology laboratories is rapidly increasing. CPDs have allowed us to perform procedures that were previously contraindicated due to high thromboembolic risk, such as in cases of intracardiac thrombosis identified at preprocedural assessment. However, several concerns related to their employment have to be defined. The selection of patients at high risk of thromboembolism is still a subjective choice of each center. The aim of this review is to update the evidence on the use of CPDs in either Cath labs or EP labs, providing an overview of their structural characteristics. Future perspectives focusing on their possible future employment are also discussed.

## 1. Introduction

The advent of cardiac transcatheter procedures (TPs) paved the way for minimally invasive approaches performed without the need for thoracotomy. Given the lower intraprocedural risk compared to cardiac surgery, these approaches allowed us to treat a lot of patients previously judged ineligible. To date, TPs have revolutionized the treatment of the most common heart diseases, such as ischemic heart diseases, valvopathies, heart failure (HF), and arrhythmias, leading to improved life expectancy, QoL, and functional status [1,2,3,4]. A number of transcatheter interventions are performed in both Catheterization labs (Cath labs) and Electrophysiology labs (EP labs) today. Percutaneous coronary interventions (PCI), transcatheter aortic valve replacement (TAVR), left atrial appendage closure (LAAC), atrial fibrillation (AF), and other arrhythmia ablations are among the most common TPs, covering approximately 90% of all interventional cardiology procedures. However, these approaches, in particular those by intracardiac or arterial route, are not free from the risk of severe complications. Among all, stroke is a well-documented and feared potential risk of TPs [5,6,7,8], posing a tremendous strain on patients, their families, and the healthcare system [9]. Subclinical neurological events or covert central nervous system infarctions are also a significant risk and are related to the development of dementia, future stroke, cognitive decline, and increased risk of mortality [10,11,12]. Procedure-related stroke or new ischemic cerebral infarctions may result from a variety of patient- and disease-related causes, such as the severity of atherosclerosis, age, gender, dyslipidemia, history of AF, HF and/or technical aspects of the procedure itself, including mechanical manipulation of instruments or interventional devices. Because of their thrombogenic nature, acute thrombus may originate at any part of endovascular catheters. Thrombus formation on transseptal sheaths despite adequate anticoagulation was reported in 9% of cases [13], as well as the thrombogenicity of guidewires [14,15]. Therefore, the thrombogenicity of endovascular catheters cannot be avoided completely in every left-sided procedure despite an ACT level > 300 s, and the risk increases in long-lasting procedures, such as ventricular (VT) tachycardia ablation. Arterial wall tissue was frequently found in the filters, accompanied by smaller amounts of calcified and necrotic core tissue. The origin of this type of debris might be the manipulation of the ablation catheter within the aortic root, ascending aorta, and aortic arch. Debris may also originate from myocardial and valve tissue by advancing and manipulating the catheters into the left ventricle via the mitral valve [16]. Apart from biological tissue, foreign material was found in the filters of patients undergoing different TPs, probably arising from hydrophilic polymer coatings used on guidewires, catheters, previously implanted ICD leads, and transseptal sheaths, which have been shown to produce clinically relevant particles [14,17,18]. New medical devices are being developed to help mitigate this risk of cardioembolic embolism during TPs. Cerebral protection devices (CPDs) are mechanical barriers designed to cover the ostium of the supra-aortic branches in the aortic arch. They are characterized by a low-profile allowing the implantation by the radial or femoral artery, filter capabilities, and stability during the procedure. Their implantation is temporary and covers the duration of the procedure, after which, they are removed. CPDs have been designed and tested in particular to reduce the cardioembolic risk during TAVR, but their use in Cath labs and EP labs is rapidly increasing. According to recent studies and meta-analyses, CPD use is safe in terms of bleeding and vascular complications, but its real effectiveness in decreasing stroke rate and other major cardiovascular embolic events is still a matter of debate [19,20,21]. Significant reduction in MACE and mortality was sometimes reported, without differences in acute kidney injury. On the contrary, significantly lower subclinical brain lesions have been detected by diffusion-weighted magnetic resonance imaging (DW-MRI) in all studies [22,23]. Data on their use in clinical practice beyond TAVR is still limited. However, there is growing evidence of CPD safety in LAAC and VT ablation with concomitant left atrial appendage (LAA) or left ventricular thrombosis [18,24]. In this review, we aimed to present the technical characteristics of current available CPDs and update clinical evidence supporting their use in Cath labs or EP labs.

## 2. Cerebral Protection Devices

To reduce the risk of stroke, CPDs have been developed to prevent debris and clots from embolizing the brain [25]. Clots can already be present at the time of the procedure or can develop during it. CPDs are usually inserted throw a radial or femoral artery access. The positioning of the device can be challenging, particularly if atherosclerotic plaques are located in the vicinity of the ostium of supra-aortic vessels or aortic arch, hampering the implantation and positioning of the device which may even promote plaque disruption and, consequently, cerebral embolization. Therefore, in patients with several risk factors for atherosclerosis, such as smoking, diabetes, obesity, and kidney disease, a preprocedural chest computed tomography angiography (CTA) may be indicated [26]. CTA can also reveal some arteriopathies, such as vascular tortuosity or aneurysms, which can preclude the use of the device or its corrected deployment. The actual efficacy of the CPDs depends on the capacity to protect the three main branches of the aortic arch and the ability of the specialists to deploy it without disrupting aortic arch plaque. They can be classified as filters or deflectors: filter devices can retain embolic material, while deflector devices reject the debris towards the descending aorta [27]. Despite deflector systems not being capable of entraping embolic material but only diverting it towards the descending aorta, no cases of embolism in inferior districts have been reported so far. There are eight types of CPDs [28]. In general, all devices are constituted by various shapes of heparin-coated polyurethane membranes of around 100 μm size pores.

### 2.1. Deflector Systems

– Embrella (Edwards Lifesciences, Irvine, CA, USA) received a European CE mark approval in 2010. It was developed to deflect embolic material during TAVR [29]. This device is inserted by right radial or brachial approach with a 6 Fr sheath. The distal end is an umbrella-like device with two heparin-coated polyurethane membranes (pore size: 100 μm). The CPD is placed through the greater curvature of the aorta, safeguarding the brachiocephalic and left common carotid artery. Since the left subclavian artery is not covered by the device, Embrella provides only partial protection to supra-aortic vessels. According to the pilot study PROTAVI-C, the device was successfully positioned in 100% of the TAVR procedures (N = 41) [30]. Although its use was associated with a reduction in lesion volume evaluated by DW-MRI, it did not prevent the occurrence of new cerebral microemboli.
– TriGuard (Keystone Heart, Caesarea, Israel) received a European CE mark in 2014 [31]. It is advanced through a 9 Fr arterial sheath placed into the left femoral artery and deployed to cover the ostia of the three supra-aortic trunks. Its new generation, the TriGuard 3, incorporates a self-expanding deflection filter composed of a structural radiopaque nitinol frame and an ultra-thin polymer mesh (nominal pore size 115 × 145 μm). The device is heparin-coated to reduce thrombogenicity and increase lubricity. The full system includes a delivery subsystem for crimping and loading the device into an 8F sheath [32]. The device was primarily developed to provide cerebral protection during TAVR [33,34]. In recent years, its use in LAAC and VT ablation procedures has rapidly increased and provided encouraging results that could pave the way for new employment in electrophysiological procedures [35,36].
– ProtEmbo CPS (Protembis, Aachen, Germany, EU) received a European CE mark in 2014. This device covers all three supra-aortic vessels, and its low-profile design provides delivery by left radial access. The heparin-coated mesh has the smallest pore size (60 μm) among all available CPDs. For this reason, it might even safeguard the cerebrum from smaller-sized debris [32,37]. The PROTEMBO C trial evaluated the safety and performance of the ProtEmbo CPS in TAVR patients [38]. The CPD met the primary safety and performance endpoints compared to prespecified historical performance goals. Enrolled patients had smaller brain lesion volumes on DW-MRI compared to prior series and no large single lesions (>150 mm^3^). The ongoing PROTEMBO SF (ClinicalTrials.gov Identifier: NCT03325283) is a prospective, observational, multicenter, intention-to-treat study of the safety and feasibility of the ProtEmbo CPS in subjects with severe symptomatic native aortic valve stenosis indicated for TAVR.

### 2.2. Filter Systems

#### 2.2.1. Supra-Aortic Filters

– Sentinel (Boston Scientific, Marlborough, MA, USA) received a European CE mark in 2014 and is the most widely used CPD so far. It is formed by a dual system filter basket containing two polyurethane mesh filters with 140 μm pores. It is advanced through a 6 Fr delivery catheter from the right radial over a 0.014 inch guidewire. It consists of a proximal filter (diameter of 9–15 mm) delivered in the brachiocephalic artery and a distal filter (diameter of 6.5–10 mm) delivered in the left common carotid artery. Through an articulating sheath, the device can be sealed into the aortic arch according to its anatomy [27]. Since the Sentinel device is deployed into supra-aortic vessels, the diameter of the supra-aortic vessels must be previously measured by CTA, because proximal and distal filters are developed to be accommodated within a brachiocephalic artery of 9 to 15 mm, and a common carotid of more than 3 mm [39]. The left vertebral artery remains unprotected. Sentinel devices have only one available size, so complete sealing might not be obtained in all aortic anatomies. Several uses of this device for LAAC and VT ablation have been reported [18,36].
– The Wirion (Abbott, Chicago, IL, USA) is a single filter usually employed for carotid stenting and lower extremity endovascular interventions [40]. It consists of a distal filter (filter basket and locking mechanism) and a rapid exchange delivery catheter. The exchange catheter has a 1.1 mm crossing profile and can be mounted on any 0.014 inch guidewire and via 6F or greater guiding catheters. The filter basket is made of a self-expanding nitinol scaffold and a nylon filter membrane with 100 μm pores. The filter can efficiently be deployed in vessels with a diameter ranging from 3.5 to 6.0 mm and at any location along the guidewire, using a proprietary remote locking system (handle at the proximal end of the delivery catheter). Since this device protects only one vessel at a time, it cannot be used alone for TPs at high risk of cardioembolism. A study reported the utility of Wirion in combination with Sentinel to complete the protection of the left vertebral artery in patients undergoing TAVR [31].
– Emblok Embolic Protection System (EPS, Innovative Cardiovascular Solutions, Grand Rapids, MI, USA) is currently only for investigational use. It is formed by an 11 F sheath device containing a 4 Fr pigtail catheter advanced through femoral access. The filter system is a 125 μm pore-size nitinol that allows the embolic filter and a radiopaque pigtail catheter to be advanced simultaneously through femoral access. It fits in various anatomies of the aorta with a diameter of up to 35 mm. The prospective, nonrandomized, multicenter, first-in-man pilot study was designed to evaluate the efficacy and safety of cerebral embolic protection utilizing the EPS-enrolled 20 patients undergoing TAVR [41]. The device was successfully placed and retrieved in all cases, and no neurological events were observed. Cerebral total new lesion volume was similar to other trials on cerebral protection during TAVR. An ongoing prospective, multicenter, single-blind, randomized controlled trial enrolling >500 patients aims to evaluate the safety, effectiveness, and performance of the EMBLOK EPS during TAVR by randomized comparison with a commercially available embolic protection device (ClinicalTrials.gov Identifier: NCT05295628).

#### 2.2.2. Full Body Filters

– Emboliner (Emboline, Santa Cruz, CA, USA) device system is currently only for investigational use. It is advanced from a 9 Fr transfemoral sheath used for the 6 Fr pigtail catheter for TAVR. It is engineered to protect all three cerebral vessels and the whole body. Early results from the SafePass 2 trial were presented in Transcatheter Cardiovascular Therapeutics 2019, reflecting no adverse events at 30 days with 100% procedural success.
– Captis (Filterlex Medical, Caesarea, Israel) is currently under development and carries a deflector mechanism with ipsilateral transfemoral access. Positioned in the aortic arch and descending aorta, it promises to provide full cerebral and body protection. The results of the prospective, single-arm, first-in-human study presented at EuroPCR 2022 involving 20 patients who underwent TAVR showed 100% technical device performance success, including deploy and retrieve and any interferences with the TAVR procedure. There were neither device-related complications nor cerebrovascular events (ClinicalTrials.gov Identifier: NCT04659538).

**Figure 1:**
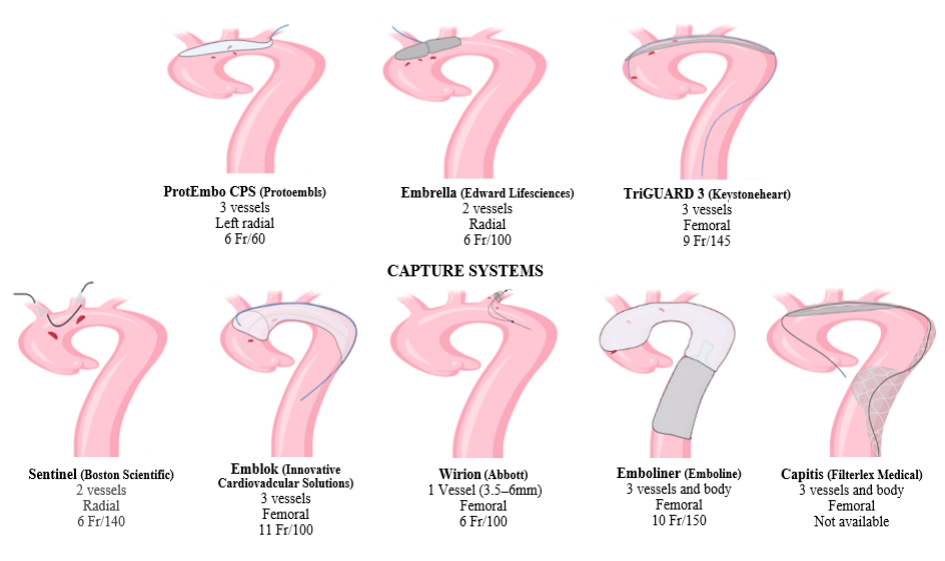
Current cerebral protection devices used in cardiovascular transcatheter procedures and main technical features (name, coverage, access site, sheath/pore size [mm]).

Figure 1 shows current CPDs used in cardiovascular TPs, while Table 1 summarizes the pros and cons of each device.

## 3. Cerebral Protection in Cath Labs

As reported above, CPDs have been designed and tested to reduce the cardioembolic risk during TAVR. In fact, during TAVR, it is hypothesized that the manipulation of large devices into the aortic arch and through the calcified native valve might mobilize and fragment atherosclerotic plaques that are prone to embolization in the cerebral circulation [44]. Structural and procedural concerns are considered the major periprocedural risk conditions [45]: aortic valve area or aortic annulus size, the degree of aortic leaflet calcification, pure aortic stenosis, high gradients, the degree of aortic atherosclerotic burden (such as porcelain aorta), as well as procedure time, repositioning of the bioprosthesis, post-dilation, the degree of anticoagulation, and the experience of the interventionalist. Ischemic stroke occurring >1 year from TAVR is named late stroke, and although its etiology is less understood, it seems to be mainly associated with patient characteristics: new-onset AF, HF, diabetes mellitus, systemic inflammatory diseases, thrombophilia, and chronic kidney disease. The disruption of the calcified native valve with denudation of endothelium and lack of endothelization of the stent-valve were also proposed among possible causes [46]. Direct evidence of embolized material in 99% of cases in dedicated trials [47] explains the high rates of peri-procedural strokes in the pivotal TAVR trials (5.5% at 30 days in intermediate-risk patients) [48]. Also, considering the expansion of TAVR to treat severe aortic stenosis in lower-risk patients [49,50], the rationale to minimize embolization and stroke is strong, and several trials tested the efficacy of different CPDs in this context, with two devices being particularly well-studied and more widely used. The Sentinel device is the most widely used CPD for cerebral protection during TAVR. As mentioned above, the whole cerebral circulation is not protected by this device, as the left vertebral artery, stemming from the left subclavian artery, is uncovered, leaving the posterior cerebral circulation prone to embolization. The Sentinel CPD has been studied extensively in earlier imaging studies and smaller trials, showing a reduction in ischemic lesions at cerebral DW-MRI (50% reduction of the number of new lesions and total lesion volume) but without a statistically significant reduction of clinical neurological events at follow-up [29]. In the larger, recent PROTECTED TAVR study [51], 3000 patients were randomly assigned to CPD or control. In this study, the incidence of peri-procedural stroke (within 72 h of TAVR) was 2.3% in the CPD group vs. 2.9% in the control group (difference: −0.6%; *p* = 0.30), thus failing to demonstrate a statistically significant advantage of CPDs. However, the incidence of disabling strokes was significantly lower in the CPD group (0.5% vs. 1.3%, difference: −0.8%; *p* < 0.05). The TriGuard 3 device offers a different mechanism of cerebral protection by deflection of debris to the lower systemic system. However, no clinical advantage has been demonstrated in the only randomized controlled trial that was prematurely halted after the commercial availability of a novel iteration of the device [42]. In the lack of clear data about the benefit of CPDs to prevent clinical neurological events, despite a strong rationale and direct biological evidence, it has been postulated that available trials were underpowered to demonstrate a reduction of clinical events individually. In a meta-analysis including only evidence from randomized controlled trials, independently of the device used, CPDs were associated with a non-significant trend towards lower risk for death or stroke (ARR: 3.5%; NNT of 28) [52]. Another more recent meta-analysis also failed to show any clinical benefit (RR for stroke: 0.88, 95% CI 0.57 to 1.36, *p* = 0.566; RR for disabling stroke: 0.85, 95% CI 0.21 to 3.41, *p* = 0.818) and also no significant difference in terms of total lesion volume on MRI was evident (−74.94, 95% CI −174.31 to 24.4, *p* = 0.139) [20]. Notably, these analyses do not include the most recent (and largest) PROTECTED TAVR study. In terms of observational evidence, an analysis of the large Society of Thoracic Surgeons-American College of Cardiology Transcatheter Valve Therapy (STS-ACC TVT) Registry database encompassing over 120,000 patients also found no significant reduction in stroke with the use of CPD, although in a propensity-match analysis, CPD was associated with a significant reduction of in-hospital stroke (odds ratio, 0.82 [95% CI, 0.69–0.97]; absolute risk difference, −0.28% [95% CI, −0.52 to −0.03]) [53]. In summary, evidence that routine use of CPDs during TAVR is of clinical benefit is lacking. However, there is a strong biological rationale and abundant proof of safety and efficacy. Therefore, it is possible that CPD might be beneficial in selected, higher-risk populations and/or in particularly young patients when maximal precautions from adverse events are warranted. Table 2 summarizes the main characteristics and results of randomized controlled trials (RCTs) investigating CPDs in TAVR. Cost-effectiveness analyses were performed only for Sentinel, reporting a probability to be cost-effective ranging from 45 to 86% at 30 days [54] and 57.5% at 5 years [55].

## 4. Cerebral Protection in EP Labs

Electrophysiologic procedures have a non-negligible risk of stroke or systemic embolism, particularly those performed on the left side of the heart. Periprocedural stroke risk is estimated between 0.1 and 0.9% in patients undergoing catheter ablation (CA) of AF [59,60,61,62], between 0.8 and 1.8% in patients undergoing CA of VT [63,64], and 0.7–1.1% in patients undergoing LAAC [65,66]. Data about the benefits of CPDs in EP labs are still missing, and those available are mainly from retrospective studies. Nevertheless, LAAC remains the second procedure with the highest use of CPD reported in the literature after TAVR. LAA is the most common site of thrombus formation in patients with nonvalvular AF [67]. Oral anticoagulation (OAC) is used to prevent and treat AF-related thrombus. However, LAA thrombus has also been noted in patients who have received full therapeutic anticoagulation [68,69]. Therefore, OAC may fail to either prevent or resolve the thrombus. In this specific scenario, LAAC may be a potential option [70,71]. Major LAAC studies have excluded patients with LAA thrombosis due to the expected high risk of systemic embolization. Consequentially, the absence of data regarding the feasibility and safety of LAAC in the presence of LAA thrombus led to a nonclear indication of this procedure in the latest guidelines on LAAC [72,73]. It must be noted that AF patients with failure of OAC therapy, including those with stroke, transient ischemic attack (TIA), or multiple findings of LAAC thrombosis at transesophageal echocardiography have limited therapeutic chances and a very high risk of incurring ischemic events [74,75]. In recent years, some retrospective studies [24] and one multicenter registry [76] highlighted the feasibility and safety of this procedure using Amulet (St. Jude Medical) and Watchman (Boston Scientific) devices. In a study, LAAC was proposed in combination with OAC as an enhancement of antithrombotic therapy in AF patients incurred in stroke/systemic embolism despite OAC, reporting optimistic results in terms of feasibility and safety after 5 years of follow-up [77]. The use of CPD in this scenario may further reduce the risk of severe intraprocedural complications. However, the use of CPD in above cited studies has been reported in <30% of procedures. In the systematic review by Sharma et al. [24], 17 patients received cerebral protection with different devices. No strokes were reported, but vascular complications were not assessed. In the multicenter TRAPEUR registry, a CPD (Sentinel) was used in five patients [76]. Procedural success was achieved in all patients, with only one major bleeding and four minor vascular complications observed. There was no periprocedural peripheral embolic event identified. More recently, a few retrospective studies focused on exploring intraprocedural and short/medium effects of CPD use in LAAC with concomitant LAA thrombosis. In the largest multicenter European study, 27 patients from eight centers with AF and LAA thrombus underwent LAA closure and cerebral protection with the Sentinel device [78]. The procedural outcome was reached in 100% of patients with any complication reported. Another single-center study treated 21 patients using Sentinel and TriGuard 3, reporting a low rate of minor vascular complications (4%) and an absence of major complications [36]. The mean procedure time, including placement of the CPD, was 103 min. Compared to LAAC without LAA sludge/thrombosis, the procedure time was longer (103 vs. 60 min, as reported in PRAGUE 17) [79]. The mean hospitalization time was 2.9 ± 2.2 days. At a follow-up of 587 days, one TIA and two non-cardiovascular deaths were noted. Currently, there are no validated criteria to identify patients with LAA thrombosis and a high risk of embolization during LAAC. Moreover, unlike TAVR, studies on the use of CMR to identify possible subclinical cerebral injuries that led to the rupture of a part of the thrombus during the device placement are lacking. Overall, despite the absence of comparative studies between use vs. nonuse of CPD in LAAC with LAA thrombosis, data available so far highlighted the feasibility and safety of this procedure, associated with either intraprocedural low risk of thromboembolism or other major complications. VTs are life-threatening arrhythmias with higher prevalence in patients with structural heart diseases [80]. In patients with HF, half of the deaths are sudden due to life-threatening ventricular arrhythmias, including VTs [81]. Frequently, VT can be difficult to manage clinically, and implantable cardioverter defibrillators (ICDs) have been shown to effectively prevent sudden cardiac death due to ventricular arrhythmias, but not to prevent the recurrence of episodes of VT. Moreover, appropriate ICD shocks are associated with significant morbidity and increased rates of mortality [82]. CA is being increasingly performed as adjunctive therapy to prevent or reduce ICD therapies when antiarrhythmic drugs are ineffective or not desired. However, VT ablation is associated with a significant risk of complications, including cerebrovascular accidents due to embolic events. The incidence of stroke in patients undergoing VT ablation in the context of structural heart disease has been reported to be up to 2.7% [83]. The presence of intra-cardiac thrombus is an absolute contraindication to VT ablation due to the high risk of embolization during the procedure. The highest prevalence of LV thrombus is related to ischemic heart diseases, while an intracardiac thrombus is rarely found in dilated cardiomyopathy. Left ventricle ejection fraction (LVEF) <40% and left ventricle (LV) aneurysm are independent predictors of LV thrombus [84]. However, 58% of patients without pre-procedural evidence of LV thrombosis undergoing routine VT ablation procedures show new cerebral ischemic lesions on postprocedural cerebral MRI [63]. Although these embolic events were initially thought to be subclinical, current investigations showed they might have negative neurocognitive effects [85]. While a number of strategies have been developed to minimize the risk of procedure-related embolic events, including peri-procedure anticoagulation, use of irrigated ablation catheters, and selective use of retrograde-aortic access, the risk of brain emboli remains high [63]. Application of the CPD during CA of VT seems to be feasible and safe, and captured debris from an acute thrombosis was demonstrated in several studies despite sufficient ACT, while foreign material was found in 55% of filters [63]. Two small studies specifically investigated CPD in patients undergoing VT ablation. A study reported feasibility and safety in a series of 11 patients with ischemic heart disease using Sentinel [18]. Debris in the device was detected in all patients at the end of the procedure. The other study reported the use of Sentinel and TriGuard 3 in seven patients undergoing VT ablation of mixed etiology without complications [35]. In a single-center experience of using CPD in EP labs, nine patients (30%) underwent VT ablation [36]. Among those, five showed LAA thrombosis, three showed LV thrombosis or severe spontaneous echo contrast, and one had mobile thrombotic material in the aortic arch detected prior to the intervention. Table 3 summarizes the clinical characteristics and main results of the above-cited studies. Paradoxically, the use of CPD during AF ablation, the most frequent cause of ischemic stroke/systemic embolism worldwide, is currently limited to one case of a patient with evidence of severe left atrial smoke [86]. A database reported a periprocedural stroke rate of 0.4% during CA of AF, which is non-negligible considering the volume of procedures performed worldwide daily [87]. Other studies reported an even higher rate, ranging from 0.9 to 1.4% [88,89]. Generation of cerebral microembolisms (firstly: air or thrombus entry via sheaths; secondly: coagulum formation on the catheter itself or over-delivered ablation lesions; thirdly: gas bubble formation occurring during CA) was reported from CMR studies, probably resulting from the technical aspects of the procedure [90]. The modality of ablation, including catheter type, affects this risk. Both preclinical and clinical studies confirmed that the air forcing into the left atrium through the septal sheath during the introduction of a ring catheter is the main source of gaseous microembolisms [91,92,93,94,95]. A pilot randomized study of the use of Sentinel for cerebral protection during CA of AF is currently ongoing (ClinicalTrials.gov Identifier: NCT04685317).

## 5. Future Perspectives

CPDs have been used for the prevention of cerebral embolization in carotid stenting procedures or cardiac surgery for nearly two decades, have a proven safety profile, and have demonstrated clinically meaningful reduction in neurological events. Now, new devices have been developed for cardiac TA. So far, CPDs are designed as filters or deflectors, with reported similar efficacy and safety in cerebral protection. However, comparative studies among the devices are not yet available.

The use of CPDs in TAVR is a developing field that recognizes the likelihood that mechanical manipulation of interventional devices in the vasculature, as well as aortic valve and aortic annulus, may result in stroke and new ischemic lesions by dislodging pre-existing atherosclerotic and other debris. Future studies with a larger population and greater statistical power are needed to prove the real benefit of CPD in preventing significant clinical cerebrovascular events in this field. Particularly, studies focusing on younger populations undergoing TAVR will be of paramount importance.

New clinical scenarios of CPD applicability should also be investigated. As the complexity of both structural and coronary interventions is constantly growing, along with treating patients at higher ischemic risk, the use of CPD could be useful, mitigating the occurrence of cerebrovascular events. Patients undergoing protected percutaneous coronary intervention with the use of impeller or complex, high-risk, and indicated percutaneous coronary interventions (CHIP) are often at increased risk of embolic events and would benefit from the use of CPD. Transcatheter interventions on the mitral valve with severe mitral annular calcification (MAC) are another source of calcium emboli, with a possible risk of severe ischemic complications [96,97]. Future studies on these unexplored fields are needed to explore the possible benefits of CPS use in different clinical scenarios. However, due to a lack of data, it is still challenging for treating clinicians to determine whether to offer CPD to all patients routinely or to use these devices selectively in patients they feel are at high risk of procedural stroke. We do not currently have randomized data to inform which patients truly are at higher risk, and who might be expected to derive benefit from CPD. Pre-procedural assessment by CT angiography, which is already used to assess the aortic root anatomy and orientation, should also be systematically used to stratify patient risk of intraprocedural stroke and then to identify patients who may benefit from the use of a CPD [98,99]. The recent availability of CPD in EP labs allowed for the treatment of patients who, in principle, would have been excluded for high intraprocedural risk of stroke, such as LAAC and VT ablation with concomitant intracardiac thrombus. Any LAAC in the presence of a thrombus runs the risk of dislodgement and embolization. Therefore, modification of the procedure to minimize interventions within the LAA has to be considered [24]. Several alternative approaches have been described so far, such as minimum LAA contrast injection and catheter manipulation (no-touch technique), removal of the delivery sheath outside the LAA with careful advancement of partially opened devices, and placement of the delivery sheath in the proximal LAA with cautious advancement. Types of LAAC devices could potentially affect the feasibility of the procedure in presence of LAA thrombus. The risk of distal touching and embolization might be higher with umbrella-shaped devices like the Watchman, because the Watchman delivery sheath has to be advanced into the LAA until its marker aligns with the ostial plane of the LAA [100]. The deployment happens from the distal to the proximal direction. The newer Watchman FLX has several new features compared with the previous generations, which may make the procedure safer [101]. The Watchman FLX has a reduced device length and closed distal nitinol loops to allow safe navigation of the partially deployed device in the LAA. In addition, when fully deployed, the Watchman FLX has only one-half the depth compared with the Watchman. On the contrary, lobe and disc devices like Amulet, which have a short length, allow for a shallow deployment without having to engage the LAA distally, and potentially avoid any contact with the thrombus. Furthermore, the deployment happens from the proximal to distal direction, with a minor risk of disturbing a distally located LAA thrombus. Partial or complete retrieval and re-deployment may significantly increase the risk of thrombus dislodgement; therefore, meticulous planning and attention to detail must be paid, including device sizing. In VT ablation, intracardiac echocardiography was demonstrated to be helpful in avoiding thrombus contact during catheter manipulation [102]. As cited above, microembolism could derive from procedure-related technical aspects; therefore, the minimum number of transeptal punctures should be used. Thrombus formation can also occur during the procedure and be dislodged into the systemic circulation in the form of microemboli. Silent cerebral lesions due to the microemboli formation during CA of AF were largely confirmed in the past [90,103], but their causal role in the development of cognitive defects in short term was reported as transient or completely absence by some studies [95,104]. Instead, the long-term effects of cerebral microembolization during AF ablation have not investigated yet and, in any case, are hard to investigate due to several confounding factors; this might be the reason why, currently, there are no studies that propose the intraprocedural use of the CPD. Few studies investigated the embolic risk of the different CA techniques [90]. RF and cryoballoon are modalities used widely throughout the world. Transcranial Doppler monitoring studies show significant microembolic signals with any ablation modality. The number of signals appears much higher with nonirrigated RF compared with irrigated RF and appears lowest—but not negligible—with cryoablation [105]. a higher degree of blood damage, platelet activation, and thrombogenesis with RF ablation compared to cryoablation [106,107]. Several studies reported that the multielectrode-phased RF (PVAC) catheter led to a substantially higher rate of microthrombi formation [108,109,110]. Managing electrodes could reduce thromboembolic events with the PVAC [111,112]. Similarly, the new very high-power, short-duration ablation (vHPSD) involving high-power (up to 90 watts) RF ablation delivered over a short duration (as little as 4 s), reported a high rate of silent cerebral lesions, albeit with no clinical strokes or cognitive impairment [113]. Pulsed-field ablation (PFA) works by using electrical fields to induce the electroporation of cells. As different cell types have different electroporation thresholds, PFA brings significant safety advantages by minimizing damage to extracardiac structures such as the esophagus and phrenic nerve. One-year outcomes from the IMPULSE and PEFCAT I + II studies found that PFA was very safe, with only 1 TIA in 121 patients, and of the eighteen patients who underwent cranial MRI scanning post-PFA ablation, one (who had suffered a clinical TIA) showed an acute lesion, and another had a single silent cerebral lesion [114]. The MANIFEST-PF survey, including data on 1758 patients across 24 clinical centers, reported TIAs in two patients (0.11%) and stroke in seven patients (0.39%) [115].

## 6. Conclusions

TA has revolutionized the treatment of the most common heart diseases, but is not free from possible severe complications. Intraprocedural stroke is a well-documented and feared potential risk of TA, despite the technological advancements and operator experience. As most cases are procedure-related embolizations, CPDs have excellent potential to prevent acute embolism, and their employment has allowed them to carry out procedures previously not feasible due to high thromboembolic risk. Given the large number of procedures performed daily in either Cath labs or EP labs, universally accepted criteria to identify patients at high risk of intraprocedural thromboembolism in whom CPD could be useful are needed. Moreover, it should be noted that the efficacy of CPD on the reduction of cerebral events has not been proved with any type of device and further adequately powered RCTs are needed to establish the optimal role of CPD in TA.

## Figures and Tables

**Table 1:** Pros and cons of current cerebral protection devices.

Device	Pros	Cons
ProtEmbo CPS [38]	Small size sheath (6 Fr); Left radial/brachial access; Mesh with the smallest pore size available; 100% successful device positioning.	Partial coverage of the supra-aortic trunk; New cerebral lesions were detected, but smaller; Available evidence only for TAVR.
Embrella [30]	Small size sheath (6 Fr); Right radial/brachial access; 100% successful device positioning.	Partial coverage of the supra-aortic trunk; New cerebral lesions were detected, but smaller; Available evidence only for TAVR.
TriGuard 3 [36,42]	Intermedium size sheath (9 Fr); Implantable through both the left and right femoral arteries; Full coverage of the supra-aortic trunk; Can be left in the aortic arch for days; 100% successful device positioning; Large amount of evidence; Available evidence for TAVR, LAAC, and VT ablation.	Femoral access; Procedural concerns if transcatheter procedure performed through the retro-aortic path; New cerebral lesions were detected, but smaller.
Sentinel [36,43]	Small size sheath (6 Fr); Right radial/brachial access; 94.4% successful device positioning; Largest amount of evidence; Available evidence for TAVR, LAAC, and VT ablation.	Partial coverage of the supra-aortic trunk; New cerebral lesions were detected but smaller.
Emblok [41]	Implantable through both the left and right femoral arteries; 100% successful device positioning.	Intermedium size sheath (11 Fr); Femoral access; Procedural concerns if transcatheter procedure performed through the retro-aortic path; New cerebral lesions were detected, but smaller; Available evidence only for TAVR.
Wirion [31]	Small size sheath (6 Fr); Right radial/brachial access; Very low amount of evidence.	Nonsufficient coverage of the supra-aortic trunk; Available evidence only for TAVR;
Emboliner	Coverage of the supra-aortic trunk and descending aorta; Implantable through both the left and right femoral arteries.	Data on the first-in-man study is not yet available.
Capitis	Coverage of the supra-aortic trunk and descending aorta; Implantable through both the left and right femoral arteries.	Data on the first-in-man study is not yet available.

**Table 2:** Main results of randomized controlled trials on cerebral protection devices in transcatheter aortic valve replacement.

RCT	Year	Sample Size	Prosthetic Valve	Endpoints	Results
DEFLECT III [56]	2015	TriGuard (*n* = 46) Controls (*n* = 39)	Balloon-expandable Self-expandable	– Safety endpoint: all-cause mortality, all stroke, life-threatening bleeding, acute kidney injury, and major vascular complications; – Efficacy endpoints: cerebral ischemic lesions on DW-MRI; neurocognitive deterioration.	– Strokes: CPD = 2, control = 2; – Safety endpoint: CPD 21.7% vs. control 30.8% (*p* = 0.34); – Efficacy endpoint: higher freedom from the ischemic lesion with CPD (46% ITT); lower new neurocognitive deterioration (CPD 3.1% vs. control 15.4%, *p* = 0.16).
EMBOL-X [57]	2015	Embol-x (*n* = 14) Controls (*n* = 16)	Balloon-expandable	– Efficacy endpoints: number of lesions on DW-MRI; lesion size.	– No strokes reported; – New lesion on DW-MRI: CPD 57% vs. control 69% (*p* = 0.70). – Smaller lesions with CPD (*p* = 0.27).
MISTRAL-C [58]	2016	Sentinel (*n* = 32) Controls (*n* = 33)	Balloon-expandable Self-expandable	– Primary endpoint: new cerebral lesions on DW-MRI; – Secondary endpoint: neurocognitive deterioration.	– Strokes: CPD = 1, control = 6; – New brain lesion: CPD 73% vs. control 87% (*p* = 0.31). – >10 new brain lesions: CPD 0% vs. control 20% (*p* = 0.03). – Smaller total lesion volume with CPD (*p* = 0.057); – Neurocognitive deterioration: CPD 4% vs. control 27% (*p* = 0.017).
CLEAN-TAVI [23]	2016	Claret Montage Dual Filter System (*n* = 50) Controls (*n* = 50)	Self-expandable	– Primary endpoint: new cerebral lesions on DW-MRI; – Secondary endpoints: difference in the volume of new lesions on DW-MRI; neurocognitive deterioration.	– Strokes: CPD = 5, control = 5; – N. of new lesions lower in the CPD group (*p* < 0.001); – The volume of new lesions is lower in the CPD group (*p* = 0.001). – Neurocognitive deterioration: no differences;
SENTINEL [43]	2017	Sentinel (*n* = 123) Controls (*n* = 119)	Balloon-expandable Self-expandable	– Safety endpoint: all-cause mortality, all stroke, acute kidney injury; – Efficacy endpoints: difference in the volume of new lesions on DW-MRI; neurocognitive deterioration.	– Stokes: CPD 5.6% vs. control 9.1% (*p* = 0.25); – Safety endpoint: CPD 7.3% vs. control 9.9% (*p* = 0.41); – Volume of new lesions: CPD 102.8 mm^3^ vs. control 178 mm^3^ (*p* = 0.33); – Neurocognitive deterioration: no differences.
REFLECT II [42]	2021	TriGuard 3 (*n* = 121) Controls (*n* = 58)	Balloon-expandable Self-expandable	– Safety endpoint: all-cause mortality, all stroke, life-threatening bleeding, acute kidney injury, major vascular complications, coronary artery obstruction, and valve-related dysfunction; Efficacy endpoints: all-cause mortality or stroke; neurocognitive deterioration; freedom from new lesions on DW-MRI; the difference in the volume of new lesions on DW-MRI.	– Strokes: CPD 8.3% vs. control 5.3% (*p* = 0.57); – Safety endpoint: CPD 15.9% vs. control 7% (*p* = 0.11); – Efficacy endpoint: neurocognitive deterioration CPD 14.1% vs. control 7.6% (*p* = 0.18); new ischemic lesions CPD 85% vs. control 84.9% (*p* = 1); similar volume of new lesions (*p* = 0.405).
PROTECTED TAVR [51]	2022	Sentinel (*n* = 1501) Controls (*n* = 1499)	Balloon-expandable Self-expandable	– Primary endpoint: clinical stroke; – Secondary endpoints: disabling stroke, death, transient ischemic attack, delirium, major or minor vascular complications at the CPD access site, and acute kidney injury.	– Primary endpoint: CPD 2.3% vs. control 2.9% (*p* = 0.30); – Secondary endpoints: disabling stroke CPD 0.5% vs. control 1.3% (*p* < 0.05); death CPD 0.5% vs. control 0.3%; transient ischemic attack CPD 0.1% vs. control 0.1%.

**Table 3:** Available studies on cerebral protection device use in EP labs.

Studies	Study Type	Year	Procedure Type	Sample Size	Results
Heeger et al. [18]	Retrospective study	2018	VT ablation	Sentinel (*n* = 11)	– Procedural success: 100%; – No strokes reported; – No complications.
Sharma et al. [24]	Systematic review	2020	LAAC with LAA thrombosis	N = 58 CPD (not specified, *n* = 17)	– Procedural success: 100%; – Strokes: 1; – Device-related thromboses: 2.
Boccuzzi et al. [78]	Registry	2021	LAAC with LAA thrombosis	Sentinel (*n* = 27)	– Procedural success: 100%; – No strokes reported; – No complications.
Zachariah et al. [35]	Research letter	2022	VT ablation	Sentinel (*n* = 6) TriGuard 3 (*n* = 1)	– Procedural success: 100%; – No strokes reported; – No complications.
Trapeur [76]	Registry	2022	LAAC with LAA thrombosis	N = 53 Sentinel (*n* = 5)	– Procedural success: 100%; – No strokes reported; – Not reported CPD safety and efficacy.
Berg et al. [36]	Retrospective study	2023	LAAC with LAA thrombosis VT ablation	Sentinel (*n* = 14) TriGuard 3 (*n* = 21) Sentinel (*n* = 5) TriGuard 3 (*n* = 4)	– Procedural success: 100%; – No strokes reported; – Four minor vascular access complications.

## Data Availability

Not requested.
